# Dual impact of information technology and dining environment: the potential mechanisms of takeout services on college student health

**DOI:** 10.3389/fpubh.2025.1476624

**Published:** 2025-02-13

**Authors:** Qi Yang

**Affiliations:** School of Innovation and Design, City University of Macau, Macau, China

**Keywords:** college students, dining environment, individual obesity, eating behavior, consumption habits

## Abstract

With the development of information technology and the popularization of the O2O business model, food delivery services have become a primary dietary choice for university students. This study, based on the social-ecological model, environmental psychology, and behavioral decision theory, aims to explore the mechanisms by which food delivery culture and campus dining environments influence obesity risk among university students, providing a basis for campus dietary management and public health policies. The study involved eight universities in Changsha, collecting 518 questionnaires on dietary behaviors, self-reported BMI, and weight change data. It also integrated data from Gaode Maps and food delivery platforms to assess the characteristics of dining environments. Multiple regression and logistic regression models were used to analyze the relationships between dining environments, food delivery frequency, and health outcomes. The results showed that food delivery frequency was significantly associated with economic status (regression coefficient = 0.418, *p* < 0.001), with students with higher living expenses being more likely to opt for food delivery. There was a significant negative correlation between healthy food delivery option scores and BMI (standardized coefficient = −0.110, *p* < 0.05), indicating that students who chose healthier food delivery options had lower BMI. Longer food delivery times (regression coefficient = −0.257, *p* = 0.052) and poorer cafeteria accessibility (regression coefficient = 0.433, *p* < 0.001) significantly increased food delivery frequency. Additionally, students in suburban universities had higher BMI (23.45 kg/m^2^) than those in urban universities (22.23 kg/m^2^), primarily due to lower availability of healthy dining options in suburban areas. The study indicates that food delivery culture, through its convenience and diversity, reinforces a tendency to consume high-calorie foods, increasing the risk of obesity. It is recommended to optimize on-campus healthy dining facilities and introduce health-focused recommendation systems on food delivery platforms to promote healthier behaviors among university students.

## 1 Introduction

With the breakthrough advancements in information technology, particularly the widespread adoption of mobile internet and online platforms, the O2O (Online-to-Offline) business model has rapidly developed globally and found broad applications across various sectors (1, 2). The O2O model refers to a process where users select or order products or services online, and then complete the consumption process offline. This model has significantly transformed the foodservice industry, including university dining services. For university students, online food delivery platforms, due to their convenience, variety, and immediacy, have become a major source of dining choices (3–6). On campuses, online food delivery platforms such as “Ele.me” and “Meituan” are highly popular among students for their convenience, variety, and fast service, gradually becoming one of the primary channels for students’ dining options. This convenience not only meets students’ daily needs but also gradually alters their eating habits and lifestyles. In particular, with the rise of food delivery culture, the obesity rate among university students has also been increasing, prompting scholars to focus on the potential health impacts of food delivery platform usage on student well-being (7).

Current research on the relationship between campus dining environments, college students’ dietary behaviors, and obesity primarily focuses on descriptive analyses of dining environments and studies on the correlation between dietary habits and obesity. Most studies have revealed significant associations between characteristics of dining environments, such as food availability (8, 9), diversity of food options (10, 11), and economic affordability (12), and the issue of obesity among college students. These studies provide valuable foundational knowledge and insights, particularly in identifying how food environments influence dietary choices and physical health. However, under the widespread popularization of food delivery culture, there remains a lack of research on the changes in campus dining environments and their mechanistic impacts on students’ specific dietary behaviors and health outcomes, particularly obesity. This gap underscores the urgent need to explore how food delivery services reshape dining environments and influence health outcomes through specific behaviors. This highlights the necessity of further investigating the mechanisms linking campus dining environments, students’ dietary behaviors, and obesity risks, to address both theoretical and empirical questions in the context of the growing prevalence of food delivery culture.

In the existing literature, research on college students’ dietary behaviors and obesity risk predominantly focuses on the physical food environment, such as the accessibility and diversity of on-campus cafeterias and off-campus restaurants, and their impacts on students’ health. However, with the rapid advancement of information technology, particularly the popularization of food delivery culture, the influence of the virtual food environment has gradually garnered attention. Although some studies have begun to explore the relationship between food delivery services and obesity, most remain at the level of descriptive analysis, lacking an in-depth examination of the underlying mechanisms. Current studies have yet to adequately investigate how food delivery services reshape college students’ dietary environments, influence their dietary behaviors, and ultimately impact health outcomes. This study addresses this gap by systematically explaining the mechanisms through which campus dining environments influence college students’ dietary behaviors and obesity, based on well-established theoretical frameworks such as the Social Ecological Model and behavioral decision-making theories in environmental psychology. The Social Ecological Model underscores the profound influence of the environment on individual behavior, analyzing the interaction between individuals and their environment across multiple levels, including individual, interpersonal, organizational, community, and macro-policy levels. This theory provides a solid foundation for this study to explain how campus dining environments shape students’ dietary behaviors and health outcomes through multi-level mechanisms (13). Environmental psychology, which examines the interplay between humans and their environment, explores how environmental features influence individual behaviors and emotions through psychological mechanisms. This framework helps us better understand how the convenience of food delivery services, alongside physical environments such as on-campus cafeterias and off-campus restaurants, alters students’ psychological perceptions and, consequently, their dietary behaviors (14). Against the backdrop of a rapidly developing food delivery culture, the convenience and immediacy of campus dining environments have been significantly enhanced, which may subtly shift college students’ dietary preferences and consumption habits, thereby exerting profound impacts on health outcomes, particularly obesity (15). Behavioral decision-making theories focus on how individuals make choices under conditions of uncertainty, particularly how external conditions influence their decisions. These theories suggest that the low cost and high availability of energy-dense foods lower the threshold for unhealthy dietary choices, while the convenience of food delivery platforms further reinforces this tendency. In this study, this framework is employed to explain how the high accessibility of food delivery services reduces students’ barriers to choosing high-calorie foods, thereby exacerbating obesity risk (16). Furthermore, traditional studies often focus on how physical environments influence students’ dietary choices through factors like accessibility and diversity. In contrast, this study expands the scope by incorporating new variables such as the convenience, temporal accessibility, and healthy options provided by food delivery platforms. More importantly, this study not only analyzes how these environmental factors directly influence students’ dietary behaviors but also examines how these factors indirectly shape their propensity to select high-calorie foods through psychological and economic mechanisms. Although existing research has pointed out the influence of dietary environments on obesity, most studies are confined to the analysis of single environmental variables. By conducting comprehensive multivariate regression analyses, this study reveals how the diversity of food delivery services and the accessibility of campus cafeterias interact in practice to jointly shape students’ health behaviors. The findings of this study offer a novel perspective and theoretical basis for managing dietary environments to reduce the obesity risk among college students.

**Figure 1:**
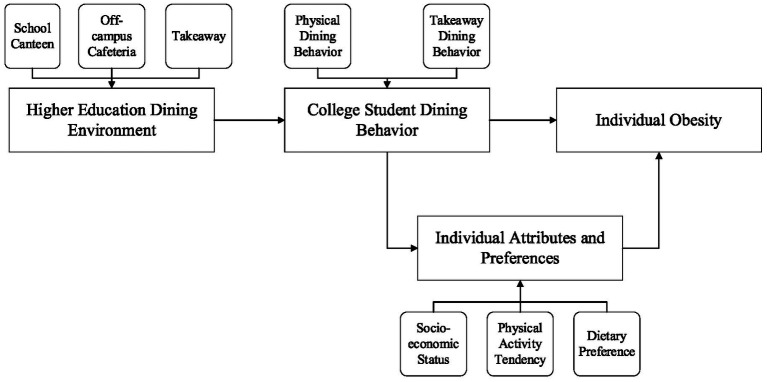
Overall framework diagram.

The overall structure of this study is arranged as follows: The first section introduces the background of university dining environments and student eating behaviors, and discusses the impact of the rise of food delivery culture on students’ dining habits and health. The second section provides a detailed description of the research methods, including data sources, variable selection, and analysis techniques. The third section presents the research results, analyzing the factors influencing individual eating behaviors and the factors related to overweight and weight gain. The fourth section discusses the significance of the research findings, with a particular emphasis on the potential impact of food delivery culture on student eating behaviors and obesity risk. Finally, the fifth section summarizes the study’s findings, outlines the limitations of the research, and proposes directions for future studies. Figure 1 illustrates the overall framework of this study, depicting the logical relationships among campus dining environments (both physical and virtual), students’ eating behaviors, and health outcomes. The study hypothesizes that the widespread adoption of food delivery culture alters the characteristics of dining environments (e.g., convenience and diversity), thereby influencing students’ eating behaviors and obesity risk.

## 2 Theoretical foundation and research hypothesis

### 2.1 Measurement of the dining environment

Urban planning focuses on the impact of urban spaces on residents’ health, particularly the role of dining environments (17). This environment is divided into two levels: macro and community. The macro level involves food supply at the national or regional scale, which is closely linked to economic factors (18). The community level, on the other hand, focuses on dining environments closely related to residents’ daily eating behaviors, such as restaurants and retail environments, and is a key area of research in urban geography and planning (19). This research field primarily evaluates dining environments based on the distribution, number, accessibility, and variety of food offerings, covering establishments such as full-service restaurants, fast food outlets, supermarkets, convenience stores, and markets (20, 21). Community dining environments are further categorized into types such as residential, workplace, and school environments. Research on residential dining environments is more common, whereas studies on school and workplace dining environments are relatively few, likely due to difficulties in obtaining research permissions. Based on the stimulus–response model in environmental psychology, the convenience and accessibility of dining environments act as external stimuli that significantly influence individuals’ food choices, subsequently altering energy intake and health outcomes (22). However, in specific contexts such as social gatherings, personal preferences become the determining factor (23).

In the preliminary data collection phase of dining environment research, researchers typically employ two main methods: one is obtaining self-reported information through interviews and surveys, and the other is directly collecting objective data using technological tools (24). In interview-based research, common tools include questionnaires, checklists, inventories, and food basket analyses. These tools help collect data on food prices, accessibility of healthy foods, and factors influencing individual choices, while also examining dining frequency, preferences, and health indicators such as BMI (25). For obtaining objective data, the dining environment is assessed by analyzing the number, proximity, and variety of restaurants and food retail outlets in specific areas, such as residences, workplaces, and schools (26). Definitions of the analysis scope vary in research, such as administrative divisions or buffers established around specific geographic locations. These buffer zones differ in size, typically ranging from 0.1 to 6 miles in radius, with 1 mile or 0.5 miles being common choices. Given the complexity of community dining environment data, selecting an appropriate statistical analysis model is crucial for accurately assessing the relationship between the dining environment and residents’ health. A literature review indicates that most studies adopt cross-sectional analysis methods, while longitudinal studies are less common. Regardless of the type, geographic analysis remains the most frequently used approach (27, 28). These studies provide a measurement framework for dining environments, especially in higher education settings, where spatial analysis is used to assess students’ dietary choices and health outcomes.

### 2.2 Relationship between dining environment and individual obesity

In many studies, individual health assessments are primarily based on overweight and obesity indicators, which are linked to the high obesity rates in Western countries and are effective in reflecting health status (29). Compared to more private data such as waist-to-hip ratio or the prevalence of chronic diseases, BMI, calculated from height and weight, is easier to obtain through self-reports. With socioeconomic development, dining out has become a trend, particularly against the backdrop of increased income levels and higher female employment rates. These dining experiences often involve foods that are high in energy, salt, fat, and sugar, while being lower in protein, dietary fiber, and micronutrients (30, 31).

Economic principles have a significant impact on food choices, with energy-dense foods such as grains, fats, and sugars being popular due to their low cost, particularly among economically disadvantaged groups. This explains why low-income populations often struggle to access affordable healthy food (16). According to behavioral decision-making theory, the low cost and high availability of energy-dense foods reduce barriers to choice, making students more likely to select fast-food outlets as dining venues, thereby increasing the risk of overweight and obesity (32). The healthiness of a community’s retail environment can be assessed by the types of food sold. The phenomenon of “food deserts” describes areas where healthy food options are scarce, which impacts residents’ health choices. Economic and geographic factors cause certain communities, particularly low-income or minority neighborhoods, to have greater exposure to energy-dense foods, exacerbating obesity issues. In developed countries such as those in Europe and North America, regions with high obesity rates often coincide with low-income or minority communities, where food desert phenomena are common (33, 34). Residents in these areas have easy access to high-calorie convenience store foods, which, like fast food, are appealing in taste, convenient to purchase, and large in portion size, leading to more severe overweight and obesity problems. In contrast, large supermarkets that provide fresh fruits, vegetables, and other healthy foods contribute to maintaining a balanced diet and reducing the risk of overweight and obesity in residents (35).

Research has found that not only the dining environment, but also lifestyle factors such as the availability of physical activity, jointly influence obesity rates. Increasing the presence of convenience stores may enhance walking opportunities in communities, helping to reduce overweight or obesity levels (36). This underscores the importance of considering multiple factors when exploring the impact of the dining environment on health, including the interactions between food accessibility, economic conditions, and lifestyle. Based on this, the following hypothesis is proposed in this study:

*H*1: Based on the socio-ecological model, the characteristics of campus dining environments significantly affect university students’ obesity risk, and this impact may be moderated by economic factors or lifestyle variables.

### 2.3 Relationship between dining environment and dining behavior

Beyond exploring the relationship between the dining environment and individual health, numerous studies focus on how the dining environment shapes individual eating behaviors. Researchers typically construct hypotheses based on nutritional theories to assess the positive and negative health impacts of specific eating habits and analyze how the dining environment influences these habits, ultimately identifying environmental types that promote healthy eating (37).

In Western societies, the widespread adoption of fast food culture is strongly associated with higher obesity rates, prompting a shift in research focus toward fast food consumption and its impacts. For instance, a study conducted in Quebec, Canada, involving 26,655 middle school students from 374 public schools, utilized Geographic Information System (GIS) technology to characterize the fast food environment around these schools. The study used the frequency of students’ junk food consumption during the previous week’s lunch period as the primary outcome measure, while controlling for covariates such as age, gender, family, and school background. A stratified logistic regression model was employed to examine the interaction between the dining environment and individual fast food consumption behavior. The study found that in areas within a 750-meter radius of schools with more than two fast food outlets, students’ frequency of junk food consumption significantly increased. In the initial analysis, the authors discussed the potential health risks associated with excessive fast food consumption, particularly the increased risk of overweight and obesity, and concluded that regulating access to fast food outlets could effectively improve students’ dietary health (38).

At the same time, Thornton and colleagues (39) also explored the relationship between the dining environment and fast food consumption, but they employed a more comprehensive assessment approach. Following expert recommendations, they classified food outlets within a selected area as either promoting health or contributing to unhealthy eating. Based on this classification, each food outlet was assigned a weight, which was used to generate a dining environment score reflecting both healthy and unhealthy aspects (FES). They then applied multilevel multinomial regression to assess the impact of these scores on the fast food purchasing behavior of 2,547 participants. Their findings concluded that in environments with healthier food options, the frequency of fast food purchases declined. These findings underscore the potential value of improving public health through the optimization of dining environments. In light of this, the present study posits the following hypothesis:

*H*2: Based on environmental psychology, campus dining environments significantly influence university students’ choice of dining methods, with these choices varying according to the convenience and accessibility of the dining environment. Specifically, the high accessibility of food delivery services tends to encourage students to use them frequently, whereas higher accessibility of off-campus restaurants and on-campus cafeterias may prompt students to choose these dining venues more often.

### 2.4 Relationship between dining behavior and individual obesity

Obesity is one of the major public health challenges facing society today, and is closely linked to many chronic diseases such as heart disease and diabetes. Globally, the prevalence of obesity is rising rapidly and has attracted widespread attention. When exploring the causes of obesity, not only genetic and environmental factors should be considered, but dining behavior is also one of the important factors affecting individual weight change, which has received more and more attention from researchers.

Dining behaviors include a variety of aspects such as choice of dining style, food type, frequency of intake, meal duration, and rate of eating, and the diversity of these behaviors has a profound impact on an individual’s energy balance and weight management. A study by Mesas et al. (40) found through a systematic assessment that the correlation between daily eating habits such as skipping breakfast and frequent intake of fast food and the risk of obesity was inconsistent, which suggests that dining behavioral research is complex to measure and define.

When viewed in detail, dining behavior is not only influenced by an individual’s physiological needs, but is also closely related to psychosocial factors. Singh’s (41) study emphasized that food is not only a basic need for survival, but also a natural reward that plays an important role in emotion regulation, social interaction, and cultural expression. People may tend to eat high-calorie, high-fat foods to seek comfort during mood swings, especially when experiencing stress, loneliness, or sadness, leading to excess energy intake, which may lead to obesity when accumulated over time. In addition, Torres and Nowson (42) found that stress is a key psychological factor that leads people to choose high-energy foods. Individuals who are chronically in a state of high stress may unconsciously increase their energy intake by eating to alleviate feelings of stress. This behavior of using food as a coping mechanism can easily lead to excess energy and weight gain if left unchecked.

Therefore, understanding and improving dietary behaviors is crucial for weight management and obesity prevention. A study by Azagba and Sharaf (43) highlighted that dietary behavior interventions may effectively reduce the risk of obesity. They emphasized that improving eating habits, such as maintaining regular meal times, choosing low-energy-dense foods, and reducing the intake of processed foods and fast food, are key strategies for controlling weight and preventing obesity. In addition, enhancing individuals’ awareness of healthy eating practices and fostering positive eating behaviors are also important approaches for effective weight management and health promotion. In light of this, the present study posits the following hypothesis:

*H*3: Based on behavioral decision-making theory, students’ dining behaviors in the campus environment—such as the frequency of using food delivery services, dining at campus cafeterias, and eating at off-campus restaurants—are significantly associated with obesity risk, with different dining choices directly influencing weight changes. Furthermore, the socio-ecological model highlights that the interaction between external environments and individual characteristics (e.g., socioeconomic status and dietary preferences) jointly impacts obesity risk. This study examines how these factors collaboratively shape students’ eating behaviors and health outcomes.

## 3 Research methodology

### 3.1 Data sources

This study comprehensively analyzes the food delivery consumption behavior of university students through a combination of questionnaire surveys and online data collection, with a particular focus on the impact of delivery culture on eating habits and health. The data sources include road network information from Gaode Maps, city building contours, POI (Points of Interest) data for restaurants near campuses, and food delivery consumption data from the Ele.me platform. This integration of data from spatial and behavioral dimensions provides a multi-layered perspective, helping to reveal the relationship between university students’ eating behaviors and food delivery culture. To ensure the universality and representativeness of the sample, this study selected higher education institutions from various locations in Changsha city as the research sites. As a core city in central China, Changsha boasts abundant university resources and a diverse food environment, making it an ideal setting for studying university students’ eating behaviors and the characteristics of the local dining environment. More importantly, Changsha’s food delivery service industry is rapidly growing, with high market penetration of multiple platforms (such as Ele.me and Meituan), offering a typical case to reflect how food delivery culture influences food choices and health among college students. In the study, higher education institutions in Changsha were categorized into urban and suburban groups based on their distance from the city center, with each group comprising four schools or campuses. To ensure the sample’s diversity and representativeness, stratified random sampling was employed. Within each school, stratified sampling was conducted according to the proportion of student numbers, ensuring that the sample could comprehensively represent students from different types of schools. A total of 70 questionnaires were distributed at each school, with 8 schools in total (4 urban and 4 suburban), resulting in 560 questionnaires being distributed. In the end, 518 valid questionnaires were collected, yielding a response rate of 92.5%. The questionnaire covered students’ eating behaviors, food preferences, food delivery frequency, BMI values, etc., aiming to comprehensively capture students’ eating habits and health status. The sample size standard was based on statistical principles for sample size calculation to ensure the robustness of multiple regression analysis and align with recommendations from existing literature. According to Cohen (44), a sample size in multiple regression should meet the standard of at least 15 to 20 samples per independent variable. The sample size selected in this study far exceeds this standard, ensuring statistical power and the robustness of the analysis results. The respondents were all full-time undergraduates or higher-level students, as they possess a high degree of autonomy in food consumption and can provide clear feedback on food delivery and eating behaviors. According to China’s physical health standards, a BMI value exceeding 23.9 is defined as overweight, and the proportion of overweight individuals in the sample was 12.8%.

Regarding online data collection, this study utilized Gaode Maps to acquire spatial data on the dining environment around campuses, including the distribution of various types of restaurants, convenience stores, and food delivery service points. Based on this spatial data, the accessibility of food delivery services and their potential impact on students’ eating behaviors were analyzed. By examining the spatial layout of the dining environment and its association with students’ food choices, the study further revealed how the dining environment influences students’ food delivery usage habits. Additionally, data from the Ele.me platform provided insights into students’ food delivery consumption behavior, including order frequency, food choices, and delivery time. This information offers important empirical support for exploring the impact of food delivery culture on university students’ eating behaviors. To ensure the accuracy and representativeness of the data, all questionnaires were distributed and collected by trained researchers, ensuring that each question accurately reflected students’ real situations. The questionnaires used a Likert five-point scale to assess students’ perceptions and attitudes toward food choices, food delivery preferences, and health status. Statistical methods were then employed to analyze the data, ensuring the scientific validity and repeatability of the results.

In terms of data analysis, this study primarily used multiple regression analysis and Logit regression analysis. Multiple regression analysis was employed to explore the relationships between the dining environment, eating behaviors, and students’ health status (such as BMI and weight changes), while Logit regression helped analyze the correlation between food delivery consumption frequency and food choices. By controlling for socioeconomic variables such as gender, age, and economic level of residence, the study ensured the robustness of the models and the reliability of the results.

### 3.2 Selection of variables

This study categorizes variables into four groups—dining environment characteristics, individual dining behaviors, individual health status, and control variables—based on literature and research hypotheses, aiming to systematically explore the relationships among dining environment, eating behaviors, and health outcomes. Dining environment characteristics include variables such as walkability satisfaction, dining accessibility, and health evaluation of dining options, intended to assess the combined impact of physical and virtual dining environments on university students. These variables are comprehensively quantified through a combination of subjective evaluations and objective data, providing a robust foundation for subsequent analysis. Individual dining behaviors primarily reflect students’ food choices and habits, with a focus on the frequency of food delivery usage, which serves as a mediating factor in analyzing the impact of dining environments on students’ health. Individual health status is measured using BMI and weight change, following the approach of Deforche et al. (45), to evaluate the direct effects of dining environments and dining behaviors on students’ health. Health data are derived from self-reported height, weight, and weight change information, facilitating quantification in regression analyses. Additionally, socioeconomic characteristics (e.g., gender, age, and living expenses) and dietary preferences (e.g., preferences for fried foods and fruits/vegetables) are incorporated as control variables to account for other potential influences on health outcomes. This approach enhances the explanatory power of the models and ensures the reliability of the results. The specific quantification methods for each variable are presented in Appendix Table A1. Subsequent analyses will construct regression models based on these variables to examine their interrelations and potential impacts on health outcomes.

### 3.3 Research model and analytical methods

This study employs multiple linear regression models and multinomial logistic regression models to analyze the impact of dining environment characteristics and individual dining behaviors on university students’ BMI and weight changes.

To analyze the relationship between continuous dependent variables (BMI and weight change) and characteristics of the dining environment as well as individual dining behaviors, a multiple linear regression model was employed. Multiple linear regression is suitable for examining the linear relationship between continuous dependent variables and multiple independent variables, effectively controlling for potential confounders and thereby revealing the independent effect of each predictor on the dependent variable. In this study, BMI and weight gain, as continuous variables, were analyzed using the multiple regression model to uncover the combined influence of dining environment characteristics and individual dining behaviors on health outcomes (46). Therefore, variables such as dining environment characteristics (e.g., walking satisfaction, delivery time, and cafeteria accessibility) and dining behavior indicators (e.g., frequency of food delivery orders) were included as independent variables, while controlling for factors such as gender, age, and living expenses. The model is expressed as:


(1) $$Y_{i}=\beta_{0}+\beta_{1}X_{1i}+\beta_{2}X_{2i}+\ldots +\beta_{k}X_{ki}+\varepsilon_{i}$$

In the equation, $Y_{i}$ represents the BMI or weight change value for the iii-th individual, $X_{1i}+X_{2i},\ldots +X_{ki}$ denotes the independent variables, which include dining environment characteristics and individual dining behaviors, and ccc represents the corresponding regression coefficients. $\beta_{k}$ estimating the regression coefficients, the significant impacts of the dining environment and individual behaviors on BMI and weight changes can be identified.

To analyze the factors influencing categorical dependent variables, a multinomial logistic regression model was utilized. Logistic regression is well-suited for scenarios where the dependent variable is categorical, allowing for the prediction of the likelihood of specific behaviors (47). The frequency of food delivery orders, as a critical indicator of individual dietary behavior, represents a multilevel categorical variable, making it appropriate for exploration using logistic regression analysis. In this study, food delivery frequency was divided into five levels, with independent variables including dining environment characteristics, individual socioeconomic attributes (e.g., gender, age, and living expenses), and dietary preferences (e.g., preferences for fried foods and fruits/vegetables). The model is structured as follows:


(2) $$\log\frac{P\left(Y=j\right)}{P\left(Y=\mathrm{base}\right)}=\alpha_{j}+\beta_{j1}X_{1i}+\beta_{j2}X_{2i}+\ldots +\beta_{jk}X_{ki}$$

In the equation, $P\left(Y=j\right)$ represents the probability that the dependent variable falls into the j-th category; $\alpha_{j}$ denotes the intercept, and $\beta_{jk}$ represents the regression coefficients of the independent variables. By estimating the model results, the relative impact of different independent variables on the frequency of takeout usage can be quantified.

## 4 Results and analysis

### 4.1 Key variable survey results

Through field visits to both physical and takeout restaurants in Changsha, it was found that the number of restaurants in the urban campus areas significantly exceeds that in suburban campuses. The spatial distribution characteristics reveal that physical restaurants are highly concentrated in central areas with a broad distribution, whereas the distribution of O2O (online-to-offline) takeout services shows a pattern of uniform dispersion followed by re-concentration, consistent with existing research findings. According to the previous definition, the healthiness of the dining environment in different campuses was evaluated. The results show that the proportion of unhealthy options in takeout restaurants ranges from 30 to 45%, while in off-campus restaurants, it ranges from 8 to 38%. The proportion of unhealthy food near suburban campuses is generally higher than that in urban areas.

Table 1 presents the survey results on the built environment, dining behaviors, and individual BMI and weight gain among the subjects from urban and suburban areas, reflecting the differences in key variables between these regions. As shown in Table 1, most university students primarily dine in campus cafeterias, with a roughly equal proportion of students ordering takeout and visiting off-campus restaurants. However, satisfaction with the taste of cafeteria food is generally low. Based on the classification of urban and suburban areas, the analysis of the survey data indicates that urban campuses score higher in walking satisfaction, accessibility to takeout services, and off-campus restaurants. Students in urban areas also use takeout services more frequently, but their BMI and weight gain rates are lower. Subsequent analyses will establish models to examine the relationships between dining environment, individual dining behaviors, BMI, and other factors, to further explore how these variables affect university students’ dining habits and health conditions.

**Table 1:** Built environment, dining behaviors, and individual BMI and weight gain in universities.

Variable Type	Variable	Description	Urban area	Suburban area
Walking satisfaction	On-campus Walking Satisfaction	1 ~ 5, 1 = Very Dissatisfied, 5 = Very Satisfied	3.82	3.44
	Off-campus Walking Satisfaction	1 ~ 5, 1 = Very Dissatisfied, 5 = Very Satisfied	3.08	2.84
Dining accessibility	Food Delivery Time	1 ~ 5, 1 = <20 min, 2 = 20 ~ 30 min; 3 = 30 ~ 40 min, 4 = 40 ~ 60 min, 5= > 1 h	2.81	3.22
	Off-campus Dining Time	1 ~ 5, 1 = <5 min, 2 = 5 ~ 10 min, 3 = 11 ~ 20 min, 4 = 21 ~ 30 min, 5= > 30 min	2.91	3.49
Individual dining behavior	Frequency of Ordering Food Delivery	1 ~ 5, 1 = Never, 2 = Once a week, 3 = 2–3 times a week, 4 = 4–5 times a week, 5 = More than 5 times a week	2.33	1.84
Individual BMI and weight gain	Current BMI	BMI = Weight / Height^2 (kg/m^2^)	22.23	23.45
	Weight Gain	Difference between current weight and weight at entry (kg)	0.46	0.51

### 4.2 Factors influencing individual dining behavior

To ensure the validity and explanatory power of the ordered Logit regression model, this study conducted a multicollinearity test. Multicollinearity among independent variables in the Logit regression model can lead to instability in the results. To detect multicollinearity, the Variance Inflation Factor (VIF) was used. A VIF value below 10 is typically considered to indicate the absence of significant multicollinearity. The results showed that the VIF values for all variables ranged between 1 and 3, maintaining a reasonable low level of multicollinearity. Additionally, the model fit was evaluated using the Hosmer-Lemeshow goodness-of-fit test, with a *p*-value of 0.23 (*p* > 0.05), indicating a good model fit and supporting the explanatory power of the independent variables on the dependent variable.

The study determined the dining preferences of university students based on their preferred dining modes—cafeterias, off-campus restaurants, or takeout—and performed a correlation analysis between the preference for cafeterias and off-campus restaurants and factors related to the physical dining environment. The results are presented in Table 2. The analysis revealed that students’ preference for off-campus restaurants was correlated with the accessibility of the restaurants, the density of nearby restaurants (indicated by the number of restaurants within walking distance), and the walking satisfaction of the surrounding area. Specifically, an increase in the density of off-campus restaurants, improved transportation accessibility, and higher walking satisfaction all contributed to a greater likelihood of students dining at off-campus restaurants. This suggests that when choosing off-campus dining options, students’ decisions are influenced by both objective and subjective environmental factors. In contrast, the preference for dining at the cafeteria was primarily positively correlated with the cafeteria’s accessibility, but showed no significant correlation with subjective environmental factors, such as walking satisfaction on campus. This indicates that as a basic food service, the cafeteria’s attractiveness is mainly influenced by its accessibility, rather than subjective environmental factors. Therefore, extended dining hours or reduced accessibility to the cafeteria would likely decrease students’ inclination to dine there.

**Table 2:** Correlation analysis.

	Variant	Correlation coefficient	*p*-value
Tendency to eat at off-campus restaurants	Number of off-campus restaurants within walking distance	−0.279***	0.000
	Off-campus walking satisfaction	0.141***	0.003
	Average time of access to off-campus cafeterias	0.088*	0.067
Tendency to eat in the cafeteria	Cafeteria meal times	0.138***	0.001
	On-campus walk satisfaction	−0.041	0.328

*, **, ***indicate *p* ≤ 0.10, *p* ≤ 0.05, *p* ≤ 0.01, respectively.

Subsequently, an ordered Logit regression model was employed to examine the relationship between the frequency of food delivery orders and factors such as the dining environment, individual socio-economic characteristics, and dietary preferences. Relevant variables were selected based on a significance level of 0.1, and the results are shown in Table 3.

**Table 3:** Logit regression analysis of factors influencing the frequency of ordering takeout.

Variable type	Explanatory variable	Ratio	*p*-value
Individual socio-economic attributes	Male = 1	−0.321*	0.081
	Female = 2	0^a^	-
	Years: number of years living in this school district	0.152**	0.024
	(a person’s) age	−0.079***	0.006
	cost-of-living standard	0.418***	0.000
Individual dietary preferences	Preference for fried food: 1 ~ 5, 1 is very dislike, 5 is very like	0.171**	0.033
	Vegetables and fruits preference: 1 ~ 5,1 for very dislike, 5 for very like	−0.374***	0.000
Catering Environmental Factors	Satisfaction with walking on campus: 1 to 5, 1 being very dissatisfied, 5 being very satisfied	−0.125	0.111
	Takeaway delivery time	−0.257**	0.052
	Takeaway taste rating: 1 ~ 5,1 is very bad, 5 is very good	0.392***	0.006
	Healthiness rating of takeaways: 1 ~ 5, 1 being very unhealthy, 5 being very healthy	0.315***	0.012
	Cafeteria accessibility	0.433***	0.000
	Off-campus cafeteria price evaluation: 1 ~ 5,1 is very unreasonable, 5 is very reasonable	−0.361***	0.008
model fit	Chi = 111.792 (*p* = 0.000), Nagelkerke = 0.220		
sample size	518		

*, **, ***indicate *p* ≤ 0.10, *p* ≤ 0.05, *p* ≤ 0.01, respectively.

As shown in Table 3, the factors influencing food delivery frequency include several individual socio-economic characteristics that significantly impact delivery frequency. Specifically, there is a noticeable difference in delivery frequency between males and females, with males (coefficient = −0.321, *p* = 0.081) showing a significantly lower tendency to order delivery compared to females. Although this result does not reach the traditional level of statistical significance, it provides preliminary evidence for the influence of gender on food delivery choices. On the other hand, the number of years an individual has lived on campus (coefficient = 0.152, *p* = 0.024) has a significant positive impact on food delivery frequency. Individuals who have lived on campus for a longer period tend to use food delivery services more frequently, as prolonged residence increases familiarity and reliance on these services, thus establishing a more fixed habit of food delivery consumption. The individual’s age (coefficient = −0.079, *p* = 0.006) has a negative impact on food delivery frequency, indicating that the frequency of food delivery decreases with age. This result reflects the changes in dietary preferences, lifestyle, and health consciousness with age, leading individuals to favor other forms of dining. Lastly, the level of living expenses positively influences food delivery frequency (coefficient = 0.418, *p* < 0.001), suggesting that individuals with higher living expenses are more likely to choose food delivery, indicating that the cost of food delivery is related to economic capacity.

In terms of individual dietary preferences, university students have a higher frequency of ordering fried foods via delivery (coefficient = 0.171, *p* = 0.033). This result indicates that individuals who prefer high-calorie, fried foods are more likely to opt for food delivery, with a higher proportion of fried foods available in delivery services. Conversely, a preference for vegetables and fruits (coefficient = −0.374, *p* < 0.001) is significantly negatively associated with delivery frequency, suggesting that individuals who favor vegetables and fruits are more likely to avoid food delivery. This is because healthy food options in delivery services are relatively limited, and vegetables and fruits are better suited for homemade meals rather than relying on delivery.

Regarding the dining environment factors, the regression analysis shows that campus walking satisfaction (coefficient = −0.125, *p* = 0.111) does not significantly affect delivery frequency. Although the coefficient is negative, the *p*-value is greater than 0.05, indicating that walking satisfaction has a weak impact on the choice of food delivery. Even with lower walking satisfaction, other dining options on campus can still meet basic dietary needs. In contrast, delivery time (coefficient = −0.257, *p* = 0.052) has a marginally significant negative effect on delivery frequency, suggesting that longer delivery times may reduce the frequency of food delivery. This highlights the importance of delivery efficiency in food delivery consumption among university students, who tend to prefer services with faster delivery times. Both delivery taste rating (coefficient = 0.392, *p* = 0.006) and delivery health rating (coefficient = 0.315, *p* = 0.012) have significant positive effects on delivery frequency. This suggests that positive evaluations of taste and health significantly increase the likelihood of students choosing food delivery, emphasizing the key role of delivery quality, especially taste and health, in students’ food delivery decisions. The accessibility of campus dining halls (coefficient = 0.433, *p* < 0.001) has a significant positive impact on food delivery frequency, indicating that individuals with easier access to campus dining halls tend to reduce their use of food delivery services. In general, the convenience and lower cost of dining halls make them a more preferred dining option. Finally, the price evaluation of off-campus restaurants (coefficient = −0.361, *p* = 0.008) has a significant negative effect on delivery frequency. This suggests that students who perceive off-campus restaurant prices as unreasonable are more likely to choose food delivery, indicating that relatively reasonable food delivery prices are an attractive factor in their consumption decisions. Thus, Hypothesis H2 is validated, as the campus dining environment significantly influences students’ dining choices. Different dining options are influenced by the convenience and accessibility of the dining environment. Specifically, high accessibility to food delivery services tends to encourage students to use them more frequently; conversely, greater accessibility to off-campus restaurants and campus dining halls may prompt students to choose these dining locations more often.

The results above indicate that the frequency of food delivery is influenced by multiple factors, including individual socio-economic background, dietary preferences, and the dining environment. In terms of individual attributes, students with higher living expenses, a preference for fried foods, and longer residence time are more likely to use food delivery services. In terms of dietary preferences, individuals who prefer healthy foods (such as vegetables and fruits) are less likely to choose food delivery. In terms of dining environment factors, delivery time, taste and health evaluations, and dining hall accessibility significantly affect delivery frequency. Based on these findings, the study shows that food delivery choices are the result of an interplay of multiple factors. Understanding these factors can help related businesses and policymakers better meet consumer needs and optimize food delivery services.

### 4.3 Factors influencing individual overweight and weight gain

**Figure 2:**
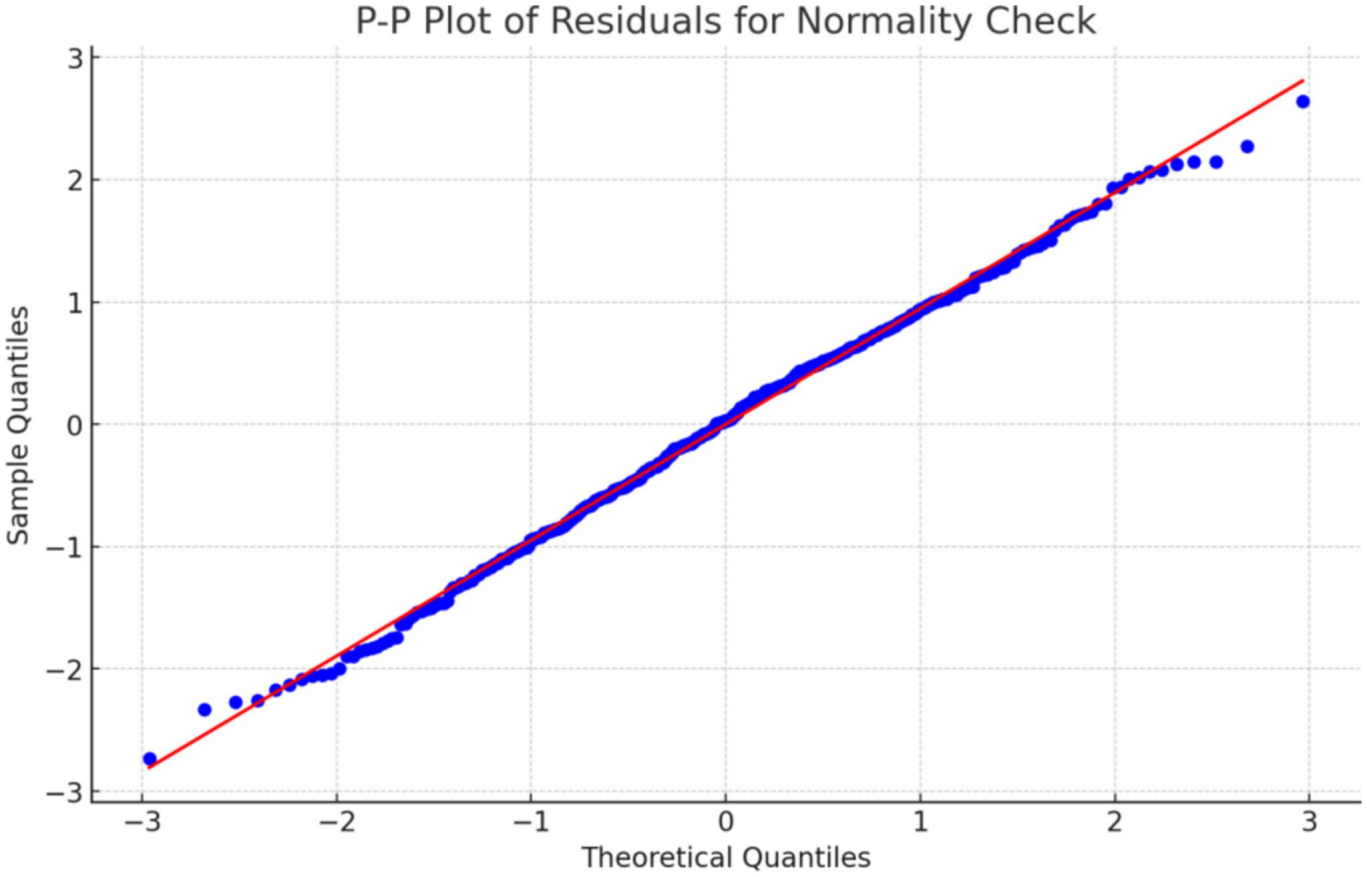
Residual normality P–P plot.

In the multiple linear regression analysis, to ensure the reliability and applicability of the model results, the main assumptions of the model were tested, including multicollinearity, residual normality, and residual independence. The Variance Inflation Factor (VIF) was used to assess the multicollinearity among the independent variables. A VIF value below 10 indicates that multicollinearity is not significant. The results showed that the VIF values ranged from 1 to 3, indicating that there is no significant multicollinearity issue among the variables, which satisfies the assumptions required for regression analysis. Residual normality is one of the fundamental assumptions of multiple linear regression. The normality of the residuals was checked using a normal probability plot (P–P plot). As shown in Figure 2, the residuals roughly follow a diagonal line, indicating that the residuals meet the normality assumption, thereby supporting the normality assumption of the regression model. Additionally, the Durbin-Watson (D-W) test was used to check for residual independence. A D-W value between 1.5 and 2.5 indicates that there is no significant autocorrelation in the residuals. The D-W value in this study was 1.98, which falls within the reasonable range, satisfying the assumption of residual independence.

In this study, non-food delivery samples were excluded, and the research scope was further narrowed to include only individuals who order food delivery. A multiple linear regression model was established with 456 samples to examine the relationship between individual dining behaviors, socio-economic characteristics, physical activity preferences, and individual BMI. In the model, “accessibility to dining halls” was recoded as a binary variable, with a value of 1 for those within a 5-min walk, and 0 otherwise. Additionally, the “types of food delivery ordered on weekdays” were categorized into three types: high healthiness (coded as 1), low healthiness (coded as −1), and other (coded as 0). The product of delivery frequency and delivery healthiness was introduced as a new independent variable in the model to reflect the total amount of healthy or unhealthy food delivery consumed by individuals. As shown in Table 4, the interaction between individual BMI and the delivery frequency-healthiness product was significantly negatively correlated (standardized coefficient = −0.110, *p* < 0.05), indicating that individuals who frequently order healthy food delivery tend to have a lower BMI. This finding aligns with previous research that suggests higher availability of healthy foods is associated with a lower risk of obesity. However, other environmental factors related to food delivery, such as the number of restaurants within the delivery range and the proportion of different types of delivery restaurants, did not show significant associations with BMI. Accessibility to dining halls also had a significant effect on BMI (standardized coefficient = −0.079, *p* < 0.10). When dining halls are easily accessible within a 5-min walk, students tend to have a lower BMI. This may be due to the higher accessibility of dining halls, which increases the convenience of dining on campus and reduces the intake of high-calorie food delivery. Thus, Hypothesis H1 is validated, as the characteristics of the campus dining environment significantly affect students’ obesity risk, with this impact potentially moderated by economic or lifestyle factors.

**Table 4:** Multiple linear regression analysis of factors influencing individual BMI.

Explanatory variable	Standardized coefficient	*p*-value
Sex: 1 = male, 2 = female	−0.423***	0.000
Weight gain: the difference between the current weight and the weight at the time of admission to the school	0.121**	0.018
cost-of-living standard	0.079*	0.084
Physical education concepts: 1 ~ 5, 1 for exercise is very unimportant, 5 for exercise is very important	−0.099**	0.045
Cafeteria 5 min reachable: 1 is 5 min walkable, 0 is not 5 min walkable	−0.079*	0.078
Frequency of takeaways * Healthiness of takeaways	−0.110**	0.029
Constant term (math.)	–	0.000
Model fit	R = 0.471^a^, R^2^ = 0.227	
Sample size	*N* = 456	

In the control variable analysis, gender, living expenses, attitude toward physical exercise, moderate-intensity exercise, dining hall accessibility, and weight change were significantly correlated with BMI. Men had a higher BMI than women, and living expenses and weight gain were positively associated with BMI. A greater emphasis on physical exercise was negatively correlated with BMI, suggesting that prioritizing physical exercise may help control weight. However, the strong correlation between moderate-intensity exercise and BMI did not provide a clear causal explanation and may only reflect a correlation rather than causality. Therefore, Hypothesis H3 is also supported, as dining behaviors in a university setting are significantly correlated with obesity risk, with different dining choices directly influencing weight changes. Furthermore, factors such as socio-economic status, physical activity preferences, and dietary habits may play important roles in the development of obesity risk.

## 5 Discussion

This study reveals the widespread prevalence of food delivery culture in university dining environments and its significant impact on students’ dietary choices and obesity risk. The results indicate that the accessibility of food delivery services increases the frequency with which students opt for delivery, particularly for high-calorie food choices. This finding highlights that the convenience of delivery platforms and the variety of food options, especially the availability of high-calorie foods, are key factors influencing students’ eating habits and weight changes. Additionally, the high accessibility of campus dining halls plays a significant role in lowering students’ BMI. The data shows that students with greater access to dining halls tend to dine on campus more frequently, resulting in significantly lower BMI values compared to those who primarily choose food delivery. This supports the conclusion that the availability of a healthy food environment can reduce obesity risk, emphasizing the important role of on-campus dining facilities in promoting student health. Further analysis also reveals that students’ socio-economic characteristics play an important moderating role in dietary choices and BMI. Specifically, students with higher living expenses have a significantly higher frequency of food delivery orders and tend to select more high-calorie foods. This reflects the potential influence of economic factors on students’ dietary choices and health risks, suggesting that while food delivery services provide convenience, their flexibility in offering unhealthy options may also pose health risks. These results shed light on how university dining environments, food delivery services, and socio-economic factors collectively impact students’ eating behaviors, offering an in-depth understanding of the complex relationship between college students’ dietary choices and health outcomes.

This study provides an in-depth exploration of the profound impact of food delivery culture on campus dining environments, uncovering how these changes influence college students’ dietary behaviors and, in turn, their obesity risk. In the existing literature, Fan et al. (8) identified a direct association between food delivery consumption and obesity, but their study primarily focused on food consumption itself, lacking an analysis of the convenience of food delivery services and its underlying mechanisms. Building on this gap, the present study adopts the multi-level framework of the Social Ecological Model, using food delivery services as an entry point to explain how campus dining environments shape students’ dietary behaviors. This study not only examines how the accessibility of food delivery services alters students’ dining preferences but also analyzes the synergistic impact of the accessibility of campus cafeterias and off-campus restaurants on students’ health, thereby enriching our understanding of how food delivery culture influences college students’ health behaviors. Moreover, Rahmawati et al. (9) highlighted the potential impact of the proliferation of food delivery services on adolescent dietary behaviors, suggesting that changes in campus dining environments may exacerbate obesity risks among student populations. Building upon this, the present study further reveals that food delivery culture, through its high convenience and diverse food options, enhances the accessibility of high-calorie diets, significantly increasing obesity risk among college students. This finding aligns with the core argument of environmental psychology, which emphasizes the profound influence of environmental characteristics on individual psychological decision-making. Additionally, this study draws on behavioral decision-making theory to elucidate key pathways in the formation of college students’ dietary behaviors. The convenience of food delivery services reduces the psychological and economic barriers to choosing high-calorie foods, encouraging irrational choices when students face time constraints or have sufficient budgets. This decision-making pattern underscores the guiding role of external environments in shaping individual behaviors, further highlighting the complex interplay between socioeconomic factors and health behaviors. The findings indicate that students with higher living allowances are more likely to opt for food delivery services, which are typically more expensive but more convenient. This observation not only underscores the importance of the economic cost of food delivery but also reveals the inducement effects of high-convenience environments on decision-making processes.

At the theoretical level, this study integrates the Social Ecological Model, environmental psychology, and behavioral decision-making theory to further expand the research framework on dietary behaviors and health risks in higher education settings. It provides a new perspective on the relationships between dietary choices, dining environments, and obesity risks among college populations. Unlike traditional studies that focus on physical dining environments, this research highlights the significance of virtual dining environments (i.e., food delivery services) in college students’ health management. It suggests that the convenience and variety of food delivery services may have a profound impact on students’ health behaviors. Additionally, the findings indicate the moderating role of socio-economic factors, such as living expenses, in the relationship between food delivery choices and health risks, further enriching the theory regarding the relationship between dietary behaviors and social attributes. This underscores the importance of considering socio-economic factors behind health behaviors. This study not only provides a new theoretical foundation for understanding how campus dining environments influence students’ health behaviors but also opens up new research directions to understand the distinct mechanisms through which virtual and physical dining environments affect health.

Based on the above findings, the following recommendations are proposed: (1) Universities should increase investments in healthy dining facilities to improve their accessibility and appeal. This can be achieved by extending dining hours, offering more healthy meal options, and optimizing the dining environment, encouraging students to choose healthier food on campus and reduce reliance on high-calorie food delivery services. Universities could also collaborate with nutrition experts to launch balanced meal menus and raise awareness among students about the importance of healthy eating through promotional campaigns. (2) Government agencies could provide clearer guidance on campus dining policies, particularly by regulating the nutritional labeling of food delivery options around campuses. They should promote the inclusion of key nutritional information, such as calories and fat content, on menus to help consumers make informed choices. Moreover, the government could support the establishment of a healthy food delivery recommendation system to promote nutritionally balanced meal options, guiding a healthier food delivery consumption trend. (3) As an essential component of virtual dining environments, food delivery platforms should take on social responsibility and play an active role in promoting healthy food choices. These platforms could create a “Healthy Eating Recommendations” section for campus areas, highlighting low-calorie and low-fat meal options. Additionally, food delivery platforms could incorporate health-related prompts on the order page, encouraging users to pair high-calorie meals with healthy drinks or vegetables, forming more balanced meal combinations. (4) For individual university students, it is recommended that universities intensify education on healthy eating and lifestyle, helping students understand the relationship between food delivery choices and health, and encouraging the development of good eating habits. This can be done through diverse activities, such as campus lectures and health challenges, to raise health awareness and guide students toward making healthier dietary choices.

Based on the findings of this study, the following recommendations are proposed from both theoretical and practical perspectives:

1. From a theoretical perspective, this study deepens the understanding of the interaction between virtual and physical food environments, filling the gap in research on the impact mechanism of food delivery culture on university students’ eating behaviors and health risks. By integrating the social-ecological model, environmental psychology, and behavioral decision theory, the study reveals how food delivery services, by lowering psychological and economic barriers, alter students’ dietary decision-making patterns. The innovation of this theoretical framework not only broadens the scope of research on food environments but also offers new insights into understanding the mechanisms underlying individual behavior in different food environments. Future research could further explore how different types of virtual food environments intersect with real-world environments to influence public health behaviors. Additionally, an in-depth analysis of the moderating role of socio-cultural contexts in shaping university students’ eating behaviors could provide theoretical guidance for promoting healthy eating habits among college students.
2. The empirical results of this study provide practical insights for optimizing health management on university campuses and improving food delivery services. The research demonstrates that the convenience of food delivery services is closely linked to students’ obesity risks, with the widespread availability of high-calorie foods on delivery platforms suggesting that promoting healthy eating options on university campuses is an effective way to mitigate obesity risks. We recommend that universities further enhance the diversity of cafeteria services by offering more healthy, low-calorie food choices, thereby reducing students’ reliance on high-calorie delivery foods. Additionally, universities could increase students’ awareness of healthy eating by introducing health-conscious labels in cafeterias and offering nutritionally balanced menus, guiding students toward healthier dietary decisions. The study also indicates that the service quality of food delivery platforms (such as delivery timeliness and food quality) significantly influences students’ frequency of food delivery consumption. This finding provides directions for optimizing food delivery services, particularly in terms of improving delivery efficiency and expanding healthy food options. Food delivery platforms can use data analytics to introduce more health-conscious meals tailored to campus populations, and implement recommendation systems to guide students toward choosing low-calorie, low-fat options, thereby fostering healthier eating behaviors. Finally, this study offers insights for public health policy. Policymakers could consider how to guide and regulate food delivery services, campus cafeterias, and surrounding dining environments to promote healthier food choices among students when developing policies related to campus nutrition and health. For instance, policies could be introduced requiring food delivery platforms to provide detailed nutritional information on food items, assisting students in making more informed dietary choices.

## 6 Conclusion

This study reveals the coupling of physical and virtual dining environments in university dining settings, highlighting both their commonalities and differences in influencing students’ dining behaviors and health. The research shows that food delivery services, as information technology-driven virtual dining environments, have a significant impact on university students’ dietary choices through temporal accessibility, but the underlying mechanisms influencing health differ significantly from those in physical dining environments (such as campus cafeterias and off-campus restaurants). The main findings of the study are as follows:

1. Campus cafeterias, as physical dining environments, are primarily influenced by spatial factors, including cafeteria accessibility and the campus walking environment. These factors determine whether students are more likely to dine on campus. In contrast, virtual food delivery services rely more on temporal accessibility, making their influence on students’ dining choices more flexible. The complementary nature of virtual and physical dining environments in shaping students’ dietary choices indicates the critical role of information technology in modern campus dining settings.
2. The frequency of food delivery consumption is a key indicator of students’ preferences and dependence on delivery services. This study found that food delivery frequency is significantly related to students’ age, duration of enrollment, and economic status, and is also influenced by dietary preferences, taste preferences for delivery food, and health awareness. This suggests that food delivery consumption is not only a convenient option but also reflects individual characteristics. However, price did not significantly impact students’ food delivery choices. There is a competitive relationship between food delivery and campus cafeterias or nearby restaurants, and improving the quality of physical dining environments could significantly reduce students’ food delivery frequency.
3. The study did not find a direct link between food delivery frequency and students’ BMI, overweight status, or weight changes. Although food delivery frequency did not directly affect BMI, students who frequently chose healthy delivery options showed a lower BMI. This indicates that the impact of food delivery services on students’ health is primarily mediated by the availability of healthy food options rather than directly contributing to obesity risk. Therefore, the variety of meals and health recommendations on delivery platforms may play an important role in shaping students’ health behaviors.
4. The results further suggest that the accessibility of healthy dining environments (e.g., convenient cafeterias) is closely related to students’ health status. BMI levels are not only influenced by food choices but also by socio-economic status, preferences for physical activity, and other factors. This highlights the need to consider these moderating factors when studying the impact of dining environments on health.

This study expands the understanding of the relationship between the campus dining environment and student health, providing scientific evidence for the management of university dining services and the formulation of public health policies. However, the study has several limitations:

1. The geographic scope of the sample in this study is relatively narrow. For reasons of data accessibility and the representativeness of Changsha in this context, the study selected students from a limited number of universities in Changsha as research participants. This may partially constrain the generalizability of the findings. Future research should broaden the geographic coverage of the sample to include universities from diverse cities and regions, thereby testing the broader applicability of the study’s conclusions.
2. This study employed a cross-sectional design and questionnaire-based survey, collecting data at a specific point in time and revealing important associations between the campus dining environment, students’ dietary behaviors, and health status. However, such a design is limited in capturing the dynamic changes in dietary behaviors and health outcomes over time and cannot establish a direct causal link between food delivery choices and obesity risk. This may lead to biases in understanding the causal chain. Furthermore, although we controlled for potential confounding factors such as individual socioeconomic attributes and dietary preferences in the model, more granular socioeconomic factors, such as income level and family background, were not further differentiated. Additionally, physical activity tendencies were measured only by students’ subjective evaluations of the importance of exercise, without distinguishing the specific effects of high-intensity versus low-intensity activities. Other variables, such as mental health, academic pressure, and the social environment of dining, which could significantly influence dietary behaviors and health outcomes, were also excluded from the scope of this study. These unmeasured variables might play crucial mediating or moderating roles between students’ dietary choices and health outcomes. Future studies could adopt longitudinal research designs to track changes in students’ dining behaviors and health indicators, thereby clarifying whether food delivery choices directly increase obesity risk or whether this is indirectly mediated by other confounding factors.
3. The questionnaire-based survey method used in this study has certain limitations in terms of the depth of data collection. Questionnaire data primarily relied on students’ self-reports, which may be subject to subjective biases and recall inaccuracies. For instance, specific details about dietary behaviors, such as the types of food ordered via delivery or the frequency of such meals, may not fully reflect actual circumstances. To improve data accuracy and reliability, future research could integrate multiple data collection methods, including observational studies, analysis of consumption records, and objective measurements of health indicators, to comprehensively uncover the profound impacts of the campus dining environment on student health.

## Data Availability

The original contributions presented in the study are included in the article/Supplementary material, further inquiries can be directed to the corresponding author.
