# Supplementary material for: Dual impact of information technology and dining environment: the potential mechanisms of takeout services on college student health
Source: Front Public Health. 2025 Feb 13;13:1476624. doi: 10.3389/fpubh.2025.1476624 (PMC11865195; doi:10.3389/fpubh.2025.1476624)
Supplement: Supplementary file 1 [file Data_Sheet_1.pdf]

Supplementary Material

Appendix A

Selection and Quantification Methods of Variables

Variable Type	Variable Name	Measurement Method	Scale Description
Dining Environment Factors	Walking Satisfaction	Questionnaire Rating (1–5)	1=Very Dissatisfied, 2=Dissatisfied, 3=Neutral, 4=Satisfied, 5=Very Satisfied
	Campus Walking Satisfaction	Questionnaire Rating (1–5)	1=Very Dissatisfied, 2=Dissatisfied, 3=Neutral, 4=Satisfied, 5=Very Satisfied
	Off-campus Walking Satisfaction	Questionnaire Rating (1–5)	1=Very Dissatisfied, 2=Dissatisfied, 3=Neutral, 4=Satisfied, 5=Very Satisfied
	Dining Accessibility	Questionnaire Rating	-
	Food Delivery Time	Time-based Rating (1–5)	1=<20 min, 2=20-30 min, 3=30-40 min, 4=40-60 min, 5=>1 hr
	Off-campus Dining Time	Time-based Rating (1–5)	1=<5 min, 2=5-10 min, 3=11-20 min, 4=21-30 min, 5=>30 min
	Food Delivery Taste Rating	Questionnaire Rating (1–5)	1=Very Poor, 2=Poor, 3=Neutral, 4=Good, 5=Excellent
	Food Delivery Healthiness Rating	Questionnaire Rating (1–5)	1=Very Unhealthy, 2=Unhealthy, 3=Neutral, 4=Healthy, 5=Very Healthy
	Canteen Accessibility	Questionnaire Rating (1–5)	1=Very Inaccessible, 2=Inaccessible, 3=Neutral, 4=Accessible, 5=Very Accessible

	Off-campus Restaurant Price Rating	Questionnaire Rating (1–5)	11=Very Unreasonable, 2=Unreasonable, 3=Neutral, 4=Reasonable, 5=Very Reasonable
Individual Dining Behavior	Food Delivery Frequency	Frequency Rating (1–5)	1=Never, 2=Once per week, 3=2-3 times per week, 4=4-5 times per week, 5=More than 5 times per week
Individual Health Indicators	Current BMI	Self-reported weight and height	Weight/Height^2 (kg/m^2)
	Weight Gain	Difference between current and initial weight (kg)	-
Control Variables	Individual Socioeconomic Attributes	-	-
	Gender	Categorical variable (1=Male, 2=Female)	-
	Years Living on Campus	Self-reported years	-
	Age	Self-reported age	-
	Living Expenses	Reported in currency amount	-

Individual Dietary Preferences	Preference for Fried Foods	Questionnaire Rating (1–5)	1=Strongly Dislike, 2=Dislike, 3=Neutral, 4=Like, 5=Strongly Like
	Preference for Fruits and Vegetables	Questionnaire Rating (1–5)	1=Strongly Dislike, 2=Dislike, 3=Neutral, 4=Like, 5=Strongly Like
